# Analogous β-Carboline Alkaloids Harmaline and Harmine Ameliorate Scopolamine-Induced Cognition Dysfunction by Attenuating Acetylcholinesterase Activity, Oxidative Stress, and Inflammation in Mice

**DOI:** 10.3389/fphar.2018.00346

**Published:** 2018-04-10

**Authors:** Shu-Ping Li, Yu-Wen Wang, Sheng-Lan Qi, Yun-Peng Zhang, Gang Deng, Wen-Zheng Ding, Chao Ma, Qi-Yan Lin, Hui-Da Guan, Wei Liu, Xue-Mei Cheng, Chang-Hong Wang

**Affiliations:** ^1^Institute of Chinese Materia Medica, Shanghai University of Traditional Chinese Medicine and The MOE Key Laboratory for Standardization of Chinese Medicines and The SATCM Key Laboratory for New Resources and Quality Evaluation of Chinese Medicine, Shanghai, China; ^2^Shanghai R&D Centre for Standardization of Chinese Medicines, Shanghai, China

**Keywords:** harmaline, harmine, scopolamine, systemic injections, memory deficits, cholinergic function, oxidative stress, inflammation

## Abstract

The analogous β-carboline alkaloids, harmaline (HAL) and harmine (HAR), possess a variety of biological properties, including acetylcholinesterase (AChE) inhibitory activity, antioxidant, anti-inflammatory, and many others, and have great potential for treating Alzheimer’s disease (AD). However, studies have showed that the two compounds have similar structures and *in vitro* AChE inhibitory activities but with significant difference in bioavailability. The objective of this study was to comparatively investigate the effects of HAL and HAR in memory deficits of scopolamine-induced mice. In the present study, mice were pretreated with HAL (2, 5, and 10 mg/kg), HAR (10, 20, and 30 mg/kg) and donepezil (5 mg/kg) by intragastrically for 7 days, and were daily intraperitoneal injected with scopolamine (1 mg/kg) to induce memory deficits and then subjected to behavioral evaluation by Morris water maze. To further elucidate the underlying mechanisms of HAL and HAR in improving learning and memory, the levels of various biochemical factors and protein expressions related to cholinergic function, oxidative stress, and inflammation were examined. The results showed that HAL and HAR could effectively ameliorate memory deficits in scopolamine-induced mice. Both of them exhibited an enhancement in cholinergic function by inhibiting AChE and inducing choline acetyltransferase (ChAT) activities, and antioxidant defense via increasing the antioxidant enzymes activities of superoxide dismutase and glutathione peroxidase, and reducing maleic diadehyde production, and anti-inflammatory effects through suppressing myeloperoxidase, tumor necrosis factor α, and nitric oxide as well as modulation of critical neurotransmitters such as acetylcholine (ACh), choline (Ch), L-tryptophan (L-Trp), 5-hydroxytryptamine (5-HT), γ-aminobutyric acid (γ-GABA), and L-glutamic acid (L-Glu). Furthermore, the regulations of HAL on cholinergic function, inflammation, and neurotransmitters were more striking than those of HAR, and HAL manifested a comparable antioxidant capacity to HAR. Remarkably, the effective dosage of HAL (2 mg/kg) was far lower than that of HAR (20 mg/kg), which probably due to the evidently differences in the bioavailability and metabolic stability of the two analogs. Taken together, all these results revealed that HAL may be a promising candidate compound with better anti-amnesic effects and pharmacokinetic characteristics for the treatments of AD and related diseases.

## Introduction

The commonest type of dementia, Alzheimer’s disease (AD), is accompanied with chronic deterioration in cognitive function and accounts for 60–70% of dementia cases (Fan and Chiu, 2014; Lu et al., 2018). According to a review in 2014, around 35.6 million people are suffering from AD at present, and the incidence of AD is progressively increasing with age and affects approximately 115.4 million people by the year of 2050 worldwide (Fan and Chiu, 2014). It is generally accepted that AD is a complicated neurodegenerative disease of multiple pathologies, which mainly associated with cholinergic transmission degeneration, oxidative stress, neuroinflammation, and so on (Coyle et al., 1983; Aucoin et al., 2005; Lin and Beal, 2006; Verri et al., 2012; Heneka et al., 2015; Bassani et al., 2017). Acetylcholinesterase (AChE) inhibitors, such as donepezil, rivastigmine and galanthamine, are preferentially prescribed for the clinical treatment of AD in the early stages (Kwon et al., 2010; Rijpma et al., 2014; Kumar and Singh, 2015). In addition, antioxidants and anti-inflammatory agents are also proverbially used in the adjuvant therapy of AD (Zhou et al., 2016; Demirci et al., 2017). However, all these agents exert limited effectiveness due to loss of efficacy gradually as the disease progress, and the medications are also associated with many toxic side effects (Zhou et al., 2016; Jeon et al., 2017; Lu et al., 2018). Namely, there is no available satisfactory treatment currently to cure AD or to alter its progressive course.

Harmaline (HAL) and harmine (HAR) are the abundant pharmacological β-carboline alkaloids of *Peganum harmala* L. with similar chemical structures (Li et al., 2017b). They possess a significant AChE, monoamine oxidase (MAO) and myeloperoxidase (MPO) inhibitory activities, antioxidant, anti-inflammatory, antitumor, and anti-hypertension effects, and can also affect the contents of various neurotransmitters then may lead to biochemical, physiological, and behavioral changes in animals and human beings (Mahmoudian et al., 2002; Frison et al., 2008; Jiménez et al., 2008; Herraiz et al., 2010; Louis et al., 2010; Bensalem et al., 2014; Wang et al., 2015; Li et al., 2017a). According to our previous studies, HAL and HAR displayed similar AChE inhibition compared to galanthamine *in vitro* (Zhao et al., 2013). Moreover, the total alkaloids (28 mg/kg) of *P. harmala* primarily contain HAL and HAR exhibited the improving effect on the deficits of learning and memory induced by scopolamine and 30% ethanol (He et al., 2015b). Subsequently, the study of He et al. (2015a) further confirmed that HAR (20 mg/kg) could ameliorate impaired memory of scopolamine-induced mice by cholinergic functions enhancement through inhibiting AChE. Nevertheless, the most recent research indicated that HAR was not only a substrate of multidrug resistance-associated protein isoform 2 (MRP2), but also had a weak metabolic stability, and eventually led to substantially lower bioavailability than that of HAL in different animals (Li et al., 2017b). Thus, it is speculated that the effective dosage of HAL against AD may be far lower than HAR. Therefore, a systematic comparison study on the anti-amnesic effects and mechanisms of the two analogs should be conducted to seek a preferable candidate compound for AD therapy.

In the current study, the modulatory effects of HAL and HAR on the scopolamine-induced memory impairments were compared using the behavioral assessment in Morris water maze (MWM). MWM has been extensively used in the study of the neurobiology and neuropharmacology of spatial learning and memory, and which plays an important role in the validation of rodent models for neurocognitive disorders such as AD (Bromley-Brits et al., 2011). Furthermore, the biochemical assays, western blotting as well as immunofluorescence analysis were comprehensively performed to clarify the possible effects of HAL and HAR on some underlying mechanisms involved in AD progressions. The results of the present study will be beneficial for evaluating the anti-amnesic effects of β-carboline alkaloids in further research and development, and could provide valuable information for clinical treatment of AD.

## Materials and Methods

### Materials

Harmaline, HAR, L-tryptophan (L-Trp), 5-hydroxytryptamine (5-HT), 5-hydroxyindole-3-acetic acid (5-HIAA), acetylcholine chloride (ACh), choline chloride (Ch), γ-aminobutyric acid (γ-GABA), L-glutamic acid monosodium salt monohydrate (L-Glu), L-phenylalanine (L-Phe), L-tyrosine (L-Tyr), theophylline [Theo, internal standard (IS)] and all other chemicals used were purchased from Sigma-Aldrich, Co., Ltd. (St. Louis, MO, United States). Scopolamine hydrobromide and donepezil hydrochloride monohydrate were purchased from TCI (Shanghai) Development, Co., Ltd. (Shanghai, China). Carboxymethylcellulose sodium (CMC-Na) and NaCl were obtained from Meilunbio^®^ Biotech, Co., Ltd. (Dalian, China). Compound titanium dioxide colorant was purchased from Shanghai Dyestuffs Research Institute, Co., Ltd. (Shanghai, China). The BCA protein quantification kit, RIPA lysis buffer, bovine serum albumin (BSA), PBST (10X), 30% acrylic amide, 10% SDS, glycine, sample loading buffer (4X) and TEMED were purchased from YEASEN Biotechnology, Co., Ltd. (Shanghai, China). Phospholipase inhibitor and protease inhibitor were purchased from Roche Applied Science (Foster City, CA, United States). PVDF membrane and Immobilon^TM^ Western chemiluminescent HRP substrate were purchased from Millipore (Billerica, MA, United States). Rabbit anti-AChE, rabbit anti-choline acetyltransferase (ChAT), rabbit anti-MPO and anti-glyceraldehyde 3-phosphate dehydrogenase (GAPDH), HRP-conjugated anti-rabbit IgG antibodies, and marker were purchased from Abcam Technology (Cambridge, MA, United States). Acetonitrile, methanol, and formic acid of HPLC grade were purchased from Fisher Scientific, Co. (Santa Clara, CA, United States). Deionized water (>18 mΩ) was purified by Milli-Q Academic System (Millipore, Corp., Billerica, MA, United States). All other chemicals were of analytical grade.

### Animals

One hundred and eight male C57BL/6 mice (aged 10 weeks) were obtained from Drug Safety Evaluation and Research Center of Shanghai University of Traditional Chinese Medicine. Mice were raised in a well-lighted air-conditioned room (25 ± 1°C) under standard environmental conditions (relative humidity: 60–65%, 12 h light–dark cycles: light on from 7:00 to 19:00) and given free access to rodent chow and tap water. All animal-use procedures were in accordance with the regulations for animal experimentation issued by the State Committee of Science and Technology of the China on 14 November 1988 and approved by the Experimental Animal Ethics Committee of Shanghai University of Traditional Chinese Medicine (No. SUTCM-2011-1107; Approval date: 10 November, 2011).

### Drug Administration

**FIGURE 1:**
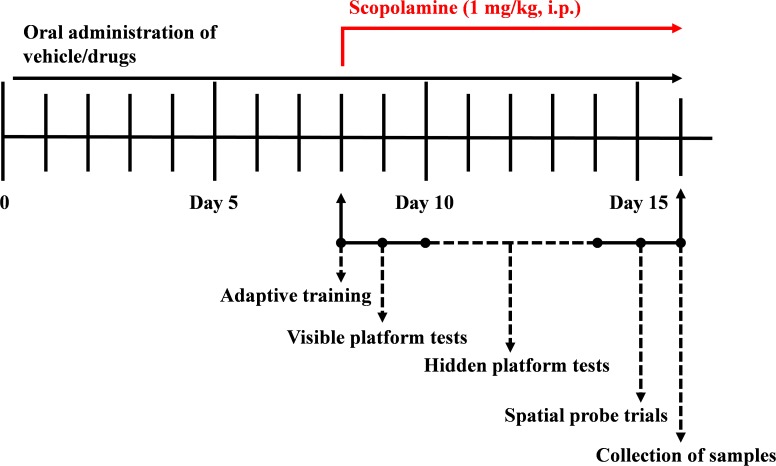
The time schedule of the experimental and treatment designs of mice.

Based on the difference in the bioavailability of HAL and HAR, the dosages were set to 2, 5, and 10 mg/kg for HAL, and 10, 20, and 30 mg/kg for HAR (Zhang, 2013; Shi et al., 2014). One hundred and eight mice were randomly divided into nine groups (12 mice per group), namely, control group (vehicle, 0.5% CMC-Na), scopolamine group (1 mg/kg), donepezil group (positive control, 5 mg/kg), HAL groups (2, 5, and 10 mg/kg, namely low: L, medium: M, and high: H dosages), and HAR groups (10, 20, and 30 mg/kg, namely L, M, and H dosages). As shown in **Figure 1**, before MWM test, the control and scopolamine groups were oral administered with 0.5% CMC-Na solution, and HAL (L, M, and H dosages), HAR (L, M, and H dosages) and donepezil (5 mg/kg) groups were administered by *gavage* for seven consecutive days. From day 8 to day 16, scopolamine (1 mg/kg) was intraperitoneally (i.p.) injected to all groups except the control group that received normal saline 30 min after the various treatments. Subsequently, the behavioral tests were daily performed 30 min after the i.p. injection of scopolamine or normal saline.

### Behavioral Assessment in Morris Water Maze

The MWM test was applied to evaluate the effects of HAL and HAR on mice spatial learning and memory as previously described by Richard Morris in 1982 with minor modifications (Morris et al., 1982; He et al., 2015a; Singh et al., 2016).

The MWM equipment consisted of a white circular water tank (diameter: 120 cm and height: 50 cm) surrounded by various visual cues (star, square, rectangle, and circle, etc.) on the pillars in fixed positions during the entire experiment. The tank was whitened by addition of non-toxic colorant, compound titanium dioxide, at 22 ± 2°C so that the platform was invisible under the water surface. The tank was virtually separated into four equal quadrants, including the Southeast, Northeast, Southwest, and Northwest. A white platform (diameter: 10 cm and height: 25 cm) was centered in the Southwest quadrant (Jeon et al., 2017; Kouémou et al., 2017; Lu et al., 2018). The whole experimental procedure was composed of adaptive training (1 day, once a day), visible platform test (1 day, four trials a day), hidden platform tests (5 days, four trials a day), and spatial probe trial (24 h after the last hidden platform test, once a day).

For the adaptive training (1 day, once a day), the first day of the MWM test (day 8 of drug treatments), each mouse was placed inside the water tank to swim for 60 s to receive an acclimatization session. The next day (day 9 of drug treatments), in order to investigate the visual, sensory, and motor of mice, the visible platform was positioned 1 cm above the water surface, and each mouse was allowed to find the platform in 60 s. The test (1 day, four trials a day) was performed prior to the hidden platform trial to screen the qualified mice. Subsequently, the platform was submerged 1 cm below the water surface in hidden platform tests (5 days, four trials a day, days 10–14 of drug treatments). The swimming activity was recorded by a video camera overhead and analyzed via a computerized tracking and image analyzer system (RD1101-MWM-G, Shanghai Mobile Datum Information Technology Company, Shanghai, China). Mice were released into the tank with head facing the tank wall from one of four quadrants successively (the Northeast, North, East, and Southeast) and allowed to find the platform in 60 s. If the mouse failed to locate the platform, it should be gently guided by the specific experimenter, and allowed to stay for 15 s in the platform. After each trial, each mouse was taken to its cage and allowed to dry under a heater. The escape latency and the escape rate were recorded for each trial. In spatial probe trial (day 15 of drug treatments), the platform was removed from the tank. Each mouse was placed into the water tank from the quadrant (for instant, the Northwest) opposite from the previous platform location (target quadrant) to receive 60 s memory retention test (Lee et al., 2016; Liu et al., 2017; Malik et al., 2017). The time in target quadrant and crossing number were recorded.

### Collection of Brain Tissues

On the 16th day after drug treatments, all the mice were anesthetized and plasma was collected by ophthalmectomy, and then brain tissues were collected after decapitation. In each group, the brains of a sub set of animals were used for immunofluorescence analysis and the others for biochemical and western blotting analysis. After sacrifices, the brain tissues were excised immediately, rinsed with normal saline, blotted surface water moisture with filter paper, and weighed. The cortex and hippocampus of each brain were divided into two parts (left and right) on ice, respectively. Some of them were fixed with 4% paraformaldehyde for immunofluorescence analysis, and the others were frozen immediately by liquid nitrogen and then stored at -80°C until analysis.

### Biochemical Assay

The cortex and hippocampus were homogenized in the ice-cold 0.1 M phosphate buffer (pH 7.4), and the 10% (w/v) homogenates were centrifuged at 4°C at 12,000 rpm for 10 min, and the supernatants were separated and then stored at -80°C for biochemical estimation (Malik et al., 2017). The levels of AChE, ChAT, superoxide dismutase (SOD), glutathione peroxidase (GSH-px), maleic diadehyde (MDA), MPO, tumor necrosis factor α (TNF-α), interleukin 1β (IL-1β), interleukin 6 (IL-6), interleukin 10 (IL-10), and nitric oxide (*NO*) in the cerebral cortex and/or hippocampus homogenates were determined by ELISA using commercially available assay kits (Jiancheng, Nanjing, China) according to the manufacturer’s protocols. Particularly, the levels of the above biochemical factors in the cerebral cortex and hippocampus were normalized using the protein concentration of each sample. Assays were performed in duplicate.

### Western Blotting Analysis

The cortex and hippocampus were homogenized in the ice-cold 50 mM Tris-HCl buffer (pH 7.4) containing phosphatase inhibitor and protease inhibitor. After centrifugation (12,000 rpm) for 10 min at 4°C, the total protein content was determined by BCA protein assay kit. The protein samples were mixed with a quarter volume of loading buffer, and then heated for 5 min at 100°C. 20 μg protein of each sample was electrophoresed by an 8% SDS-PAGE and transferred to PVDF membranes. Afterward, the membrane was blocked for 1 h by 5% non-fat milk at room temperature and incubated with anti-GAPDH (1:5000), anti-AChE (1:1000), anti-ChAT (1:1000), and anti-MPO (1:1000) at 4°C overnight. Subsequently, the membranes were thoroughly rinsed with PBST and then incubated with HRP-conjugated anti-rabbit (1:5000) secondary antibody for 2 h at room temperature. Following completely washing with PBST, the protein bands were visualized with ECL prime kit.

### Immunofluorescence Analysis

Myeloperoxidase generation was visualized by immunofluorescence microscopy as described previously with minor changes (Gray et al., 2008). After fixing in 4% paraformaldehyde, the cortices were cut into 5 μm thick sagittal sections, and were deparaffinized and processed for immunofluorescence analysis. Brain sections were placed in a citric acid (pH 6.0) solution for antigen repair in a microwave oven. Subsequently, the autofluorescence quenching was carried out for 5 min. After blocking in 2% BSA, the sections were probed with anti-MPO (1:100) overnight at 4°C. Afterward, the sections were washed with PBS (pH 7.4) for three times and incubated with HRP-conjugated anti-rabbit (1:300) secondary antibody for 30 min at room temperature. Then, 4′,6-diamidino-2-phenylindole (DAPI) was employed for DNA counterstaining and the brain sections were observed under a fluorescence microscope (Nikon ECLIPSE C1, Japan), and the images were captured accordingly. Here, the fluorescence of DAPI or MPO was measured at an excitation wavelength of 330–380 or 510–560 nm and an emission wavelength of 420 or 590 nm, respectively.

### Effects of HAL and HAR on the Neurotransmitters

#### Extraction Procedure

A rapid, reliable and convenient precipitation method was applied to prepare the cerebral cortex homogenates and plasma samples. A 100 μL sample was added with 200 μL ice-cold acetonitrile containing 60 ng/mL IS in a 1.5 mL centrifuge tube and vortex-mixed for 1 min. The mixture was then centrifuged at 12,000 rpm for 10 min (4°C). A 5 μL aliquot of the supernatant from each sample was injected into ultra performance liquid chromatography combined with electrospray ionization (ESI) quadrupole tandem mass spectrometry (UPLC-ESI-MS/MS) for quantitative analysis.

#### UPLC-ESI-MS/MS Analysis

The concentrations of various neurotransmitters were simultaneous quantified with SHIMADZU LC-30AD UPLC system (Shimadzu, Kyoto, Japan) connected to an AB Sciex QTRAP^®^ 6500 triple quadrupole mass spectrometer (SCIEX, United States) equipped with an ESI source using positive ion detection mode for multiple reaction monitoring. Chromatographic separation was conducted using a ZIC-cHILIC column (150 mm × 2.1 mm, 3 μm, Merck-Sequant, Germany) with a SeQuant ZIC-cHILIC guard column (20 mm × 2.1 mm, 5 μm, Merck-Sequant, Germany). The mobile phase, acetonitrile-water (65:35, v/v) containing 0.1% formic acid was delivered at a flow rate of 0.2 mL/min. To protect the mass spectrometer from contaminations, the flow was diverted to waste during the first minute after injection. All other instrumental parameters were set according to our previous study (Jiang, 2016). The UPLC-ESI-MS/MS method was well-validated and was successfully applied to determine the concentrations of neurotransmitters in the cerebral cortex homogenates and plasma (Jiang, 2016).

### Statistical Analysis

Statistical evaluation was carried out with SPSS version 18.0 software, and the data were expressed as the mean ± SD. The presented escape latency and path length data in MWM test were analyzed by repeated measures two-way ANOVA. The other behavioral data and the biomarkers changes *in vitro* were tested by one-way ANOVA for multiple comparisons. Significant results were marked according to conventional critical *P*-values: ^#^*P* < 0.05; ^##^*P* < 0.01; ^###^*P* < 0.001, *vs.* the control group. ^∗^*P* < 0.05; ^∗∗^*P* < 0.01; ^∗∗∗^*P* < 0.001, *vs.* the scopolamine-induced group.

## Results

### HAL and HAR Attenuated Cognitive Dysfunction of Scopolamine-Treated Mice in MWM

**FIGURE 2:**
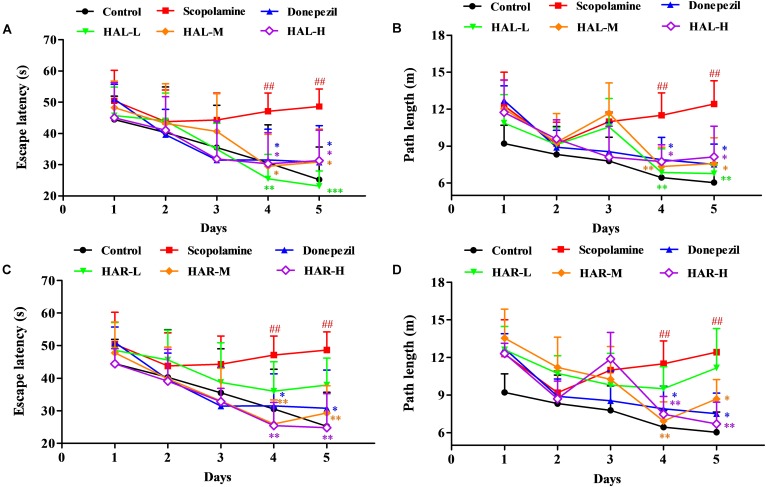
The effects of HAL (2, 5, and 10 mg/kg, namely low: L, medium: M, and high: H dosages) and HAR (10, 20, and 30 mg/kg, namely L, M, and H dosages) on scopolamine-induced memory impairments mice. HAL (L, M, and H dosages), HAR (L, M, and H dosages), and donepezil (5 mg/kg) were administered by *gavage* for 7 days prior to the training for Morris water maze (MWM) tests, and continued during the testing. Memory impairments were induced by scopolamine (1 mg/kg, i.p.). The escape latency **(A)** and path length **(B)** of HAL (L, M, and H dosages) treated groups in hidden platform tests for five consecutive days, and the escape latency **(C)** and path length **(D)** of HAR (L, M, and H dosages) treated groups in hidden platform tests for five consecutive days. The error bar (SD) for data points in this figure was not given in order to clearly discriminate the difference among treatments. *N* = 12/group. ^##^*P* < 0.01, *vs.* the control group. ^∗^*P* < 0.05; ^∗∗^*P* < 0.01; ^∗∗∗^*P* < 0.001, *vs.* the scopolamine-induced group.

To determine the effects of HAL (L, M, and H dosages) and HAR (L, M, and H dosages) on spatial learning and memory, the MWM task was carried out in the scopolamine-induced memory impairment mice. Two-way repeated measures analysis with mixed model showed that the latency to reach the platform was significantly different among the experimental groups of mice (*F*_(8,396)_ = 26.011, *P* < 0.001) and days of acquisition training (*F*_(4,396)_ = 15.274, *P* < 0.001). There was no significant interaction between groups and days (*F*_(32,396)_ = 1.132, *P* > 0.05), suggesting that the differences among groups were dependent on the treatment. As presented in **Figure 2**, in the hidden platform testing, the escape latency and path length in scopolamine-induced group were significantly longer than those of the control group on the fourth (**Figure 2A**, *F*_(8,99)_ = 3.135, *P* < 0.01; **Figure 2B**, *F*_(8,99)_ = 4.074, *P* < 0.01) and fifth days (**Figure 2A**, *F*_(8,99)_ = 4.164, *P* < 0.01; **Figure 2B**, *F*_(8,99)_ = 4.548, *P* < 0.01), indicating the successfully-constructed mice model of memory impairment. However, the scopolamine-induced cognitive deficits were reversed by both HAL and HAR. Compared with scopolamine-induced group, the escape latency and path length were effectively reduced in HAL (L, M, and H dosages) and HAR (M and H dosages) treated groups on the fourth (**Figure 2A**, *F*_(8,99)_ = 4.975, *P* < 0.01; *F*_(8,99)_ = 2.816, *P* < 0.05; *F*_(8,99)_ = 2.795, *P* < 0.05; **Figure 2B**, *F*_(8,99)_ = 3.836, *P* < 0.01; *F*_(8,99)_ = 3.231, *P* < 0.01; *F*_(8,99)_ = 2.658, *P* < 0.05; **Figure 2C**, *F*_(8,99)_ = 4.673, *P* < 0.01; *F*_(8,99)_ = 4.881, *P* < 0.01; **Figure 2D**, *F*_(8,99)_ = 3.817, *P* < 0.01; *F*_(8,99)_ = 3.129, *P* < 0.01) and fifth days (**Figure 2A**, *F*_(8,99)_ = 12.637, *P* < 0.001; *F*_(8,99)_ = 2.802, *P* < 0.05; *F*_(8,99)_ = 2.772, *P* < 0.05; **Figure 2B**, *F*_(8,99)_ = 4.014, *P* < 0.01; *F*_(8,99)_ = 2.722, *P* < 0.05; *F*_(8,99)_ = 2.531, *P* < 0.05; **Figure 2C**, *F*_(8,99)_ = 4.574, *P* < 0.01; *F*_(8,99)_ = 4.632, *P* < 0.01; **Figure 2D**, *F*_(8,99)_ = 2.547, *P* < 0.05; *F*_(8,99)_ = 3.905, *P* < 0.01). Howbeit, when treated with the low dosage of HAR at 10 mg/kg (L dosage group), no significantly decrease in the escape latency and path length throughout the training period on the fourth (**Figure 2C**, *F*_(8,99)_ = 1.182, *P* > 0.05; **Figure 2D**, *F*_(8,99)_ = 0.494, *P* > 0.05) and fifth days (**Figure 2C**, *F*_(8,99)_ = 0.873, *P* > 0.05; **Figure 2D**, *F*_(8,99)_ = 0.201, *P* > 0.05). It also can be seen from **Figure 2**, the escape latency and path length of donepezil-treated (5 mg/kg) were markedly shortened compared with those of the scopolamine-induced group on the fourth (**Figure 2A**, *F*_(8,99)_ = 2.668, *P* < 0.05; **Figure 2B**, *F*_(8,99)_ = 2.643, *P* < 0.05) and fifth days (**Figure 2A**, *F*_(8,99)_ = 2.780, *P* < 0.05; **Figure 2B**, *F*_(8,99)_ = 2.897, *P* < 0.05) during training trial session three.

**FIGURE 3:**
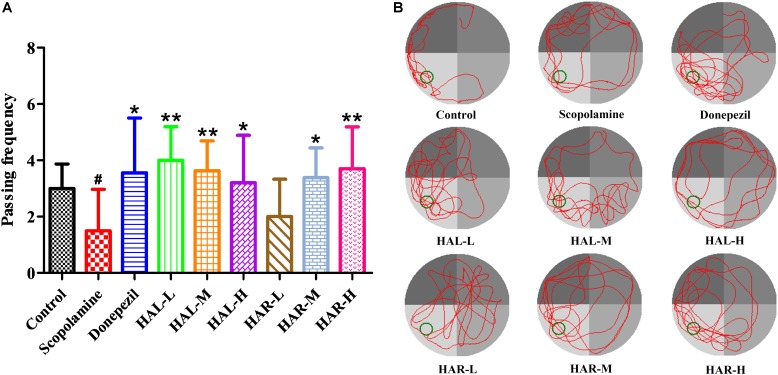
The effects of HAL (2, 5, and 10 mg/kg, namely low: L, medium: M, and high: H dosages) and HAR (10, 20, and 30 mg/kg, namely L, M, and H dosages) on scopolamine-induced memory impairments mice. The frequency of mice passing through the platform location **(A)**, and the swimming tracks of mice in water tank **(B)**. The green circle indicates the location of the hidden platform, while the red curves represent the tracks of mice movement. *N* = 10/group. ^#^*P* < 0.05, *vs.* the control group. ^∗^*P* < 0.05; ^∗∗^*P* < 0.01; *vs.* the scopolamine-induced group.

On the day after the hidden platform testing, the spatial probe trial was conducted by taking the platform away to evaluate the spatial memory of all mice. As shown in **Figure 3**, the passing frequency was remarkably declined in the scopolamine-induced group (*F*_(8,81)_ = 5.498, *P* < 0.05), illustrating that 1 mg/kg of scopolamine damaged the formation of spatial memory in mice. Particularly, apart from HAR (L dosage) treatment group (*F*_(8,81)_ = 0.494, *P* > 0.05), the other treatment groups of HAL (L, M, and H dosages) and HAR (M and H dosages) dramatically raised the passing frequency (*F*_(8,81)_ = 11.667, *P* < 0.01; *F*_(8,81)_ = 9.072, *P* < 0.01; *F*_(8,81)_ = 4.507, *P* < 0.05; *F*_(8,81)_ = 7.063, *P* < 0.05; *F*_(8,81)_ = 8.583, *P* < 0.01, respectively), and the increase of HAL-L dosage group was more notable compared to the other groups (**Figure 3**). Besides, donepezil-treated (5 mg/kg) group also significantly increased the passing frequency in comparison with the scopolamine-induced mice (**Figure 3**, *F*_(8,81)_ = 5.345, *P* < 0.05). All these results suggested that HAL (L, M, and H dosages) and HAR (M and H dosages) could ameliorate the spatial memory ability of scopolamine-induced cognitive dysfunction mice, and HAL exhibited stronger effects than HAR on the spatial learning and memory in MWM.

### Effects of HAL and HAR on Scopolamine-Induced Cholinergic System, Oxidative Stress, and Inflammation

**FIGURE 4:**
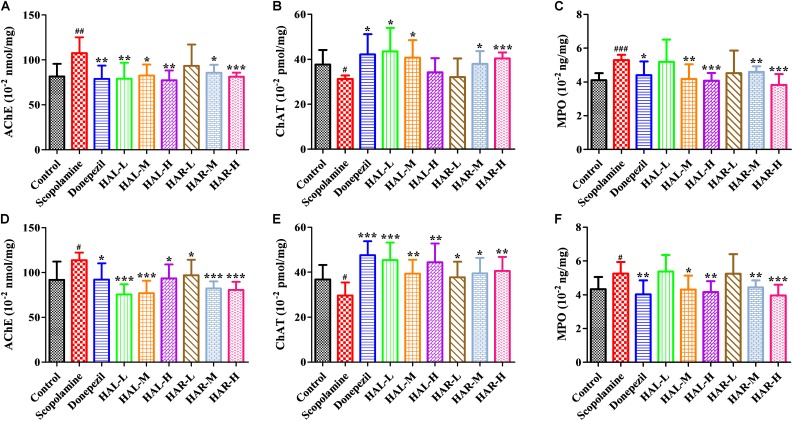
The effects of HAL (2, 5, and 10 mg/kg, namely low: L, medium: M, and high: H dosages) and HAR (10, 20, and 30 mg/kg, namely L, M, and H dosages) on the activities of acetylcholinesterase (AChE), choline acetyltransferase (ChAT), and myeloperoxidase (MPO) in the cerebral cortex **(A–C)** and hipocampus **(D–F)** of scopolamine-induced cognitive impairments mice, respectively. Data were expressed as the mean ± SD (*n* = 10). ^#^*P* < 0.05; ^##^*P* < 0.01; ^###^*P* < 0.001, *vs.* the control group. ^∗^*P* < 0.05; ^∗∗^*P* < 0.01; ^∗∗∗^*P* < 0.001, *vs.* the scopolamine-induced group.

**FIGURE 5:**
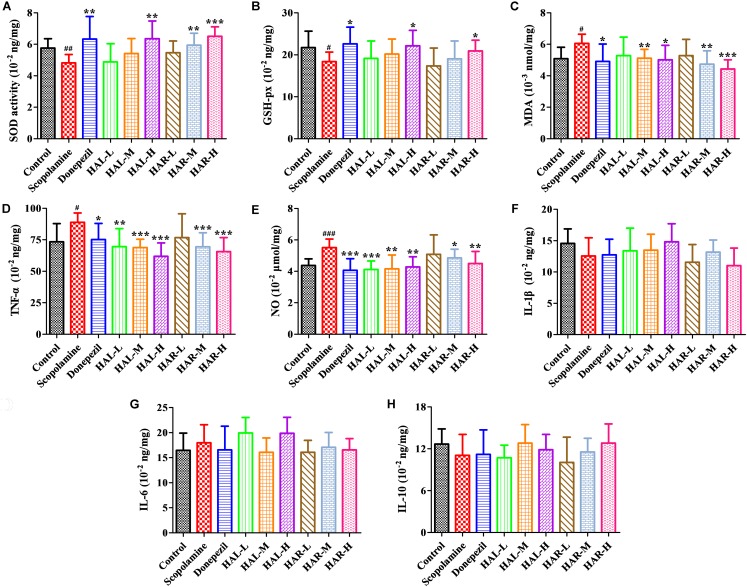
The effects of HAL (2, 5, and 10 mg/kg, namely low: L, medium: M, and high: H dosages) and HAR (10, 20, and 30 mg/kg, namely L, M, and H dosages) on the activity or production of superoxide dismutase (SOD) **(A)**, glutathione peroxidase (GSH-px) **(B)**, maleic diadehyde (MDA) **(C)**, tumor necrosis factor α (TNF-α) **(D)** and nitric oxide (*NO*) **(E)** in the cerebral cortex of scopolamine-induced cognitive impairments mice, respectively. Data were expressed as the mean ± SD (*n* = 10). ^#^*P* < 0.05; ^##^*P* < 0.01; ^###^*P* < 0.001, *vs.* the control group. Interleukin 1β (IL-1β) **(F)**, interleukin 6 (IL-6) **(G)** and interleukin 10 (IL-10) **(H)**. ^∗^*P* < 0.05; ^∗∗^*P* < 0.01; ^∗∗∗^*P* < 0.001, *vs.* the scopolamine-induced group.

**FIGURE 6:**
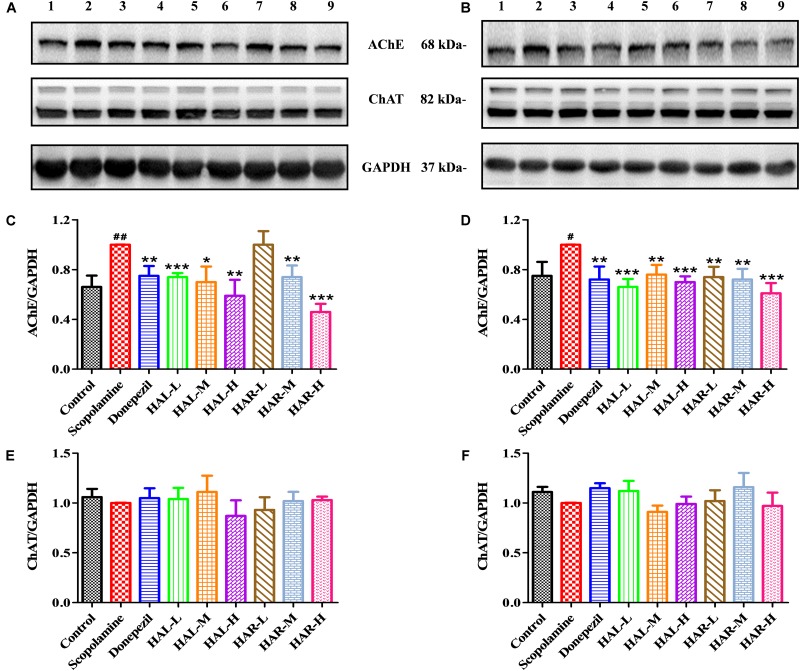
The effects of HAL (2, 5, and 10 mg/kg, namely low: L, medium: M, and high: H dosages) and HAR (10, 20, and 30 mg/kg, namely L, M, and H dosages) on the protein expression levels of acetylcholinesterase (AChE), and choline acetyltransferase (ChAT) in the cerebral cortex **(A,C,E)** and hipocampus **(B,D,F)** of scopolamine-induced cognitive impairments mice. Data were expressed as the mean ± SD (*n* = 3). ^#^*P* < 0.05; ^##^*P* < 0.01, *vs.* the control group. ^∗^*P* < 0.05; ^∗∗^*P* < 0.01; ^∗∗∗^*P* < 0.001, *vs.* the scopolamine-induced group.

**FIGURE 7:**
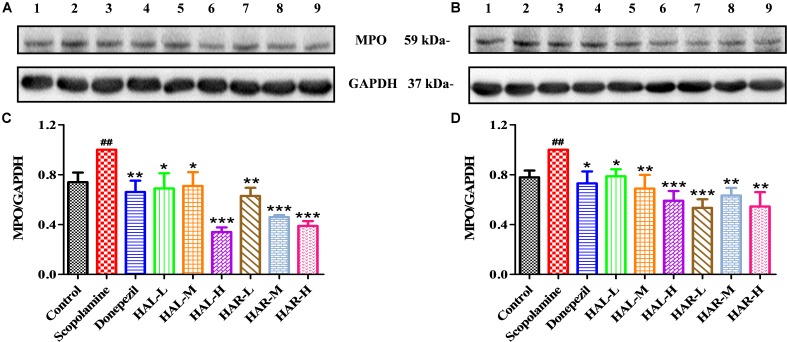
The effects of HAL (2, 5, and 10 mg/kg, namely low: L, medium: M, and high: H dosages) and HAR (10, 20, and 30 mg/kg, namely L, M, and H dosages) on the protein expression levels of myeloperoxidase (MPO) in the cerebral cortex **(A,C)** and hipocampus **(B,D)** of scopolamine-induced cognitive impairments mice. Data were expressed as the mean ± SD (*n* = 3). ^#^*P* < 0.05; ^##^*P* < 0.01, *vs.* the control group. ^∗^*P* < 0.05; ^∗∗^*P* < 0.01; ^∗∗∗^*P* < 0.001, *vs.* the scopolamine-induced group.

**FIGURE 8:**
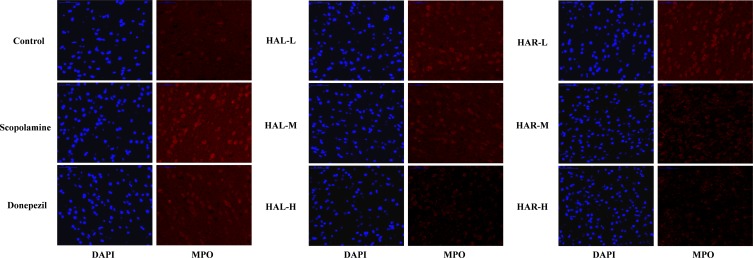
The effects of HAL (2, 5, and 10 mg/kg, namely low: L, medium: M, and high: H dosages) and HAR (10, 20, and 30 mg/kg, namely L, M, and H dosages) on the generation of myeloperoxidase (MPO) in the cerebral cortex of scopolamine-induced cognitive impairments mice visualized by immunofluorescence microscopy.

To further elucidate the potential mechanisms of HAL and HAR in improving memory induced by scopolamine, the levels of multifarious biochemical factors and protein expressions associated with the cholinergic system, oxidative stress, and inflammation were investigated. **Figures 4**, **5** present the changes of various biochemical factors in the cortex or hippocampus, and **Figures 6**–**8** give the effects of HAL and HAR on the protein expressions in the cortex of mice.

### Effects of HAL and HAR on Cholinergic Deficits

To clarify the effects of HAL and HAR on the cerebral cholinergic function, the changes in the activities and protein levels of AChE and ChAT in the cortical and hippocampal tissues of all mice were assessed. As shown in **Figures 4A,D**, treatment with scopolamine at 1 mg/kg significantly increased the activity of AChE in the cortex and hippocampus (*F*_(8,81)_ = 10.553, *P* < 0.01; *F*_(8,81)_ = 6.177, *P* < 0.05). Except HAR-L dosage group in the cortex, the other HAL (L, M, and H dosages) and HAR (M and H dosages) treatment groups considerably reduced the AChE activity (**Figure 4A**, *F*_(8,81)_ = 10.837, *P* < 0.01; *F*_(8,81)_ = 8.621, *P* < 0.05; *F*_(8,81)_ = 15.177, *P* < 0.01; *F*_(8,81)_ = 8.615, *P* < 0.05; *F*_(8,81)_ = 16.330, *P* < 0.001; **Figure 4D**, *F*_(8,81)_ = 46.593, *P* < 0.001; *F*_(8,81)_ = 34.179, *P* < 0.001; *F*_(8,81)_ = 7.968, *P* < 0.05; *F*_(8,81)_ = 4.769, *P* < 0.05; *F*_(8,81)_ = 49.802, *P* < 0.001; *F*_(8,81)_ = 46.665, *P* < 0.001). Likewise, donepezil-treated group evidently lessened the activities of AChE compared with the levels in the scopolamine-induced group (**Figure 4A**, *F*_(8,81)_ = 13.138, *P* < 0.01; **Figure 4D**, *F*_(8,81)_ = 7.400, *P* < 0.05). Additionally, the scopolamine-induced group displayed a significant decrease in the activity of ChAT in both cortex and hippocampus (**Figure 4B**, *F*_(8,81)_ = 5.481, *P* < 0.05; **Figure 4E**, *F*_(8,81)_ = 4.874, *P* < 0.05). Nevertheless, HAL (L and M dosages) and HAR (M and H dosages) administrations appreciably increased the ChAT activity in the cortex (**Figure 4B**, *F*_(8,81)_ = 7.956, *P* < 0.05; *F*_(8,81)_ = 8.390, *P* < 0.05; *F*_(8,81)_ = 7.835, *P* < 0.05; *F*_(8,81)_ = 51.927, *P* < 0.001), and the ChAT levels of all HAL and HAR treatment groups in the hippocampus were significantly higher than those in the scopolamine-induced group (**Figure 4E**, *F*_(8,81)_ = 18.139, *P* < 0.001; *F*_(8,81)_ = 9.665, *P* < 0.01; *F*_(8,81)_ = 14.113, *P* < 0.01; *F*_(8,81)_ = 5.178, *P* < 0.05; *F*_(8,81)_ = 7.599, *P* < 0.05; *F*_(8,81)_ = 11.953, *P* < 0.01), and the effect of HAL-L dosage group on the ChAT activity was more significant in comparison with HAR administration groups.

As depicted in **Figure 6**, compared to the control group, the protein expression levels of AChE in cerebral cortex and hippocampus of scopolamine-induced group were prominently increased (**Figure 6C**, *F*_(8,18)_ = 36.632, *P* < 0.01; **Figure 6D**, *F*_(8,18)_ = 14.823, *P* < 0.05), while the protein levels of ChAT were almost unchanged (**Figures 6E,F**). Following treatments with HAL (L, M, and H dosages), HAR (M and H dosages) and donepezil, except for HAR (L dosage), the protein levels of AChE in the cortex were reduced markedly compared with scopolamine-induced group (**Figure 6C**, *F*_(8,18)_ = 50.590, *P* < 0.001; *F*_(8,18)_ = 16.950, *P* < 0.05; *F*_(8,18)_ = 30.993, *P* < 0.01; *F*_(8,18)_ = 23.821, *P* < 0.01; *F*_(8,18)_ = 52.196, *P* < 0.001; *F*_(8,18)_ = 29.284, *P* < 0.01), while the levels of ChAT were not altered (**Figure 6E**). And similar effects on the levels of AChE and ChAT were seen with HAL and HAR (L, M, and H dosages) administrations in the hippocampus (**Figure 6D**, *F*_(8,18)_ = 41.195, *P* < 0.001; *F*_(8,18)_ = 28.111, *P* < 0.01; *F*_(8,18)_ = 47.611, *P* < 0.001; *F*_(8,18)_ = 30.539, *P* < 0.01; *F*_(8,18)_ = 33.225, *P* < 0.01; *F*_(8,18)_ = 40.032, *P* < 0.001; **Figure 6F**). In addition, the inhibitory effects of HAR on the AChE protein expression were dose-dependent, while HAL had a notable inhibitory effect at low dose of 2 mg/kg (**Figure 6**). Thus, HAL and HAR treatments prevented against scopolamine-induced elevation of the AChE activity and protein expression and reduction of the ChAT activity, which demonstrated that HAL and HAR could enhance the function of cholinergic system, and alleviate the behavioral dysfunction. In particular, HAL manifested a significant improvement under low dosage.

### Effects of HAL and HAR on Oxidative Stress Markers

The effects of HAL and HAR on the activities of antioxidant enzymes SOD, GSH-px, and the level of MDA in the cortex were measured. As **Figure 5** indicated, the scopolamine-induced group visibly attenuated the SOD and GSH-px activities compared to the control group (**Figure 5A**, *F*_(8,81)_ = 12.254, *P* < 0.01; **Figure 5B**, *F*_(8,81)_ = 4.577, *P* < 0.05). Whereas, HAL (H dosage), HAR (M and H dosages), and donepezil administrations significantly increased the SOD activity (**Figure 5A**, *F*_(8,81)_ = 13.287, *P* < 0.01; *F*_(8,81)_ = 13.424, *P* < 0.01; *F*_(8,81)_ = 34.510, *P* < 0.001; *F*_(8,81)_ = 8.670, *P* < 0.01), and HAL, HAR (both H dosages) and donepezil treatments clearly raised the activity of GSH-px (**Figure 5B**, *F*_(8,81)_ = 6.010, *P* < 0.05; *F*_(8,81)_ = 6.008, *P* < 0.05; *F*_(8,81)_ = 6.860, *P* < 0.05). Furthermore, with HAL, HAR (both M and H dosages) and donepezil (5 mg/kg) treatments, the significant attenuation of MDA level increase was observed compared with the scopolamine-induced mice (**Figure 5C**, *F*_(8,81)_ = 9.302, *P* < 0.01; *F*_(8,81)_ = 6.565, *P* < 0.05; *F*_(8,81)_ = 12.690, *P* < 0.01; *F*_(8,81)_ = 33.627, *P* < 0.001; *F*_(8,81)_ = 5.874, *P* < 0.05). These results suggested that HAL and HAR may prevent against scopolamine-induced oxidative damage through protecting the cortex from oxidative stress. Noticeably, the oxidative damage could be improved only at high dosages for both HAL and HAR, and the improvements of HAL and HAR were comparable in general.

### Effects of HAL and HAR on Inflammation Levels

The effects of HAL and HAR on the activities of inflammatory factors MPO, TNF-α, IL-1β, IL-6, IL-10 and the level of *NO* in the cortex or hippocampus were analyzed. The activities of MPO (cortex and hippocampus) and TNF-α (cortex) were elevated in the scopolamine-induced mice compared to the control mice (**Figure 4C**, *F*_(8,81)_ = 34.740, *P* < 0.001; **Figure 4F**, *F*_(8,81)_ = 6.729, *P* < 0.05; **Figure 5D**, *F*_(8,81)_ = 6.316, *P* < 0.05), but the levels of IL-1β, IL-6, and IL-10 were not changed (**Figures 5F–H**). However, HAL, HAR (both M and H dosages) and donepezil administrations resulted in a significant reduction of the MPO activity in the cortex and hippocampus in a dose dependent manner (**Figure 4C**, *F*_(8,81)_ = 10.435, *P* < 0.01; *F*_(8,81)_ = 32.309, *P* < 0.001; *F*_(8,81)_ = 16.397, *P* < 0.01; *F*_(8,81)_ = 31.291, *P* < 0.001; *F*_(8,81)_ = 7.129, *P* < 0.05; **Figure 4F**, *F*_(8,81)_ = 7.212, *P* < 0.05; *F*_(8,81)_ = 10.533, *P* < 0.01; *F*_(8,81)_ = 9.150, *P* < 0.01; *F*_(8,81)_ = 18.903, *P* < 0.001; *F*_(8,81)_ = 11.641, *P* < 0.01), and HAL (L, M, and H dosages), HAR (M and H dosages) and donepezil treatments also weaken the activity of TNF-α in the cortex (**Figure 5D**, *F*_(8,81)_ = 10.377, *P* < 0.01; *F*_(8,81)_ = 28.257, *P* < 0.001; *F*_(8,81)_ = 31.088, *P* < 0.001; *F*_(8,81)_ = 15.966, *P* < 0.001; *F*_(8,81)_ = 23.530, *P* < 0.001; *F*_(8,81)_ = 5.909, *P* < 0.05). In addition, apart from HAR-L dosage group, a noticeable attenuation of *NO* level increase was found in the other HAL, HAR, and donepezil treatment mice compared with the scopolamine-induced amnesia mice (**Figure 5E**, *F*_(8,81)_ = 21.319, *P* < 0.001; *F*_(8,81)_ = 12.996, *P* < 0.01; *F*_(8,81)_ = 11.911, *P* < 0.01; *F*_(8,81)_ = 5.227, *P* < 0.05; *F*_(8,81)_ = 8.799, *P* < 0.01; *F*_(8,81)_ = 17.349, *P* < 0.001), and the inhibitory effects of HAL on TNF-α and *NO* were more pronounced than those of HAR.

As show in **Figure 7**, compared with the control mice, the protein expression levels of cortical and hippocampal MPO in scopolamine-induced mice were elevated (**Figure 7C**, *F*_(8,18)_ = 29.975, *P* < 0.01; **Figure 7D**, *F*_(8,18)_ = 32.014, *P* < 0.01). Following administrations of donepezil, HAL, and HAR (L, M, and H dosages), the protein expressions of MPO were apparently dropped in the cortex and hippocampus compared with scopolamine-induced group. Besides, the results of immunofluorescence determination also revealed that HAL and HAR treatments could strikingly reduce the MPO expression of the cortex with a dose-dependent manner, and the inhibitions of HAL and HAR were comparable (**Figure 8**). All these findings implied that HAL and HAR could abate the scopolamine-induced inflammatory injury through inhibiting the cortex and hippocampus from inflammation.

### Modulator Effects of HAL and HAR on the Neurotransmitters of Scopolamine-Treated Mice

**FIGURE 9:**
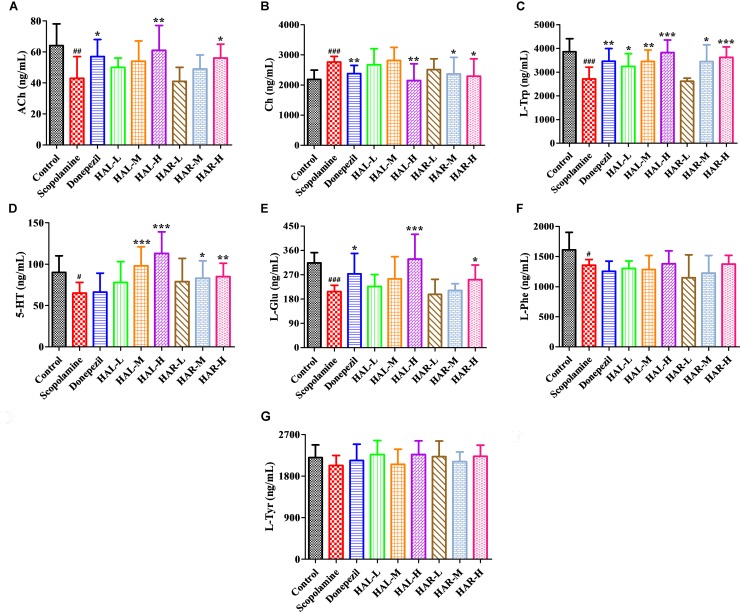
The effects of HAL (2, 5, and 10 mg/kg, namely low: L, medium: M, and high: H dosages) and HAR (10, 20, and 30 mg/kg, namely L, M, and H dosages) on the contents of neurotransmitters in the plasma of scopolamine-induced cognitive impairments mice. *N* = 10/group. ACh **(A)**, Ch **(B)**, L-Trp **(C)**, 5-HT **(D)**, L-Glu **(E)**, L-Phe **(F)**, L-Tyr **(G)**. ^#^*P* < 0.05; ^##^*P* < 0.01; ^###^*P* < 0.001, *vs.* the control group. ^∗^*P* < 0.05; ^∗∗^*P* < 0.01; ^∗∗∗^*P* < 0.001, *vs.* the scopolamine-induced group.

**FIGURE 10:**
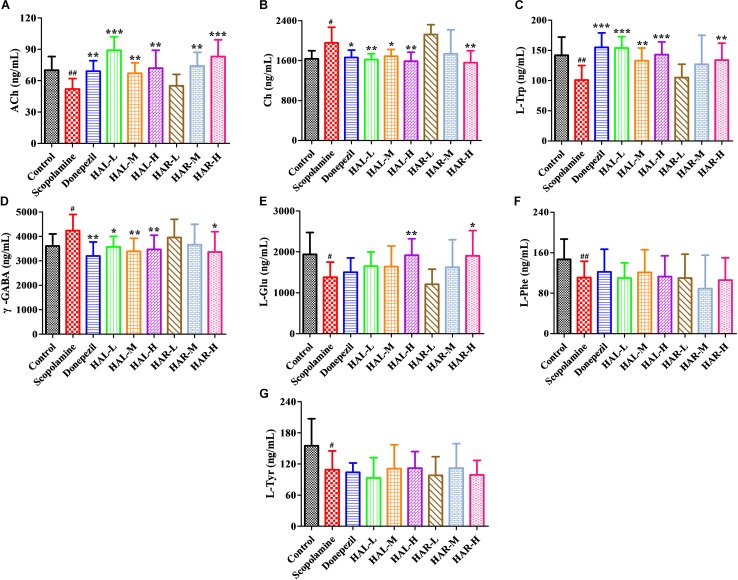
The effects of HAL (2, 5, and 10 mg/kg, namely low: L, medium: M, and high: H dosages) and HAR (10, 20, and 30 mg/kg, namely L, M, and H dosages) on the contents of neurotransmitters in the cerebral cortex of scopolamine-induced cognitive impairments mice. *N* = 10/group. ACh **(A)**, Ch **(B)**, L-Trp **(C)**, γ-GABA **(D)**, L-Glu **(E)**, L-Phe **(F)**, L-Tyr **(G)**. ^#^*P* < 0.05; ^##^*P* < 0.01, *vs.* the control group. ^∗^*P* < 0.05; ^∗∗^*P* < 0.01; ^∗∗∗^*P* < 0.001, *vs.* the scopolamine-induced group.

Various plasmatic and cortical neurotransmitters were estimated using UPLC-ESI-MS/MS and the alterations are presented in **Figures 9**, **10**, respectively. Based on the method for determination of neurotransmitters established previously, the contents of ACh, Ch, L-Trp, 5-HT, L-Glu, L-Phe, L-Tyr in the plasma and ACh, Ch, L-Trp, γ-GABA, L-Glu, L-Phe, L-Tyr in the cortex were above the lower limit of quantitation, and then all of them were successfully quantified.

After induction of scopolamine at the dosage of 1 mg/kg, the concentrations of ACh, L-Trp, 5-HT, L-Glu, and L-Phe were decreased in the plasma (**Figure 9A**, *F*_(8,81)_ = 11.453, *P* < 0.01; **Figure 9C**, *F*_(8,81)_ = 23.137, *P* < 0.001; **Figure 9D**, *F*_(8,81)_ = 7.359, *P* < 0.05; **Figure 9E**, *F*_(8,81)_ = 55.531, *P* < 0.001; **Figure 9F**, *F*_(8,81)_ = 6.330, *P* < 0.05), whereas with no impact on L-Tyr (**Figure 9G**, *F*_(8,81)_ = 2.091, *P* > 0.05). However, when treatments with HAL and HAR at high dosage, the contents of ACh and L-Glu were elevated (**Figures 9A,E**), and the contents of L-Trp were evidently raised with HAL (L, M, and H dosages) and HAR (M and H dosages) administrations in a dose-dependent manner (**Figure 9C**). Moreover, the HAL and HAR (M and H dosages) treated mice exhibited a significant elevation of 5-HT level compared to the scopolamine-induced mice (**Figure 9D**). But, the HAL and HAR administrations did not modulate the contents of L-Phe and L-Tyr (**Figures 9F,G**). Besides, HAL (H dosage) as well as HAR (M and H dosages) produced an effect on reducing the content of Ch in the plasma (**Figure 9B**).

Furthermore, **Figure 10** shows the variations of the neurotransmitters in the cerebral cortex. Following inducing of scopolamine, the contents of ACh, L-Trp, L-Glu, L-Phe, and L-Tyr were obviously decreased (**Figure 10A**, *F*_(8,81)_ = 11.296, *P* < 0.01; **Figure 10C**, *F*_(8,81)_ = 10.979, *P* < 0.01; **Figure 10E**, *F*_(8,81)_ = 7.216, *P* < 0.05; **Figure 10F**, *F*_(8,81)_ = 10.340, *P* < 0.01; **Figure 10G**, *F*_(8,81)_ = 6.834, *P* < 0.05), while the contents of Ch and γ-GABA were significantly raised in the cortex compared to the control group (**Figure 10B**, *F*_(8,81)_ = 7.377, *P* < 0.05; **Figure 10D**, *F*_(8,81)_ = 5.580, *P* < 0.05). Nevertheless, the contents of ACh were observably elevated in HAL (L, M, and H dosages) and HAR (M and H dosages) groups, and the contents of L-Trp were notably raised in HAL (L, M, and H dosages) and HAR (H dosage) groups, and the amounts of L-Glu were also elevated at the high dosage of HAL and HAR groups (**Figures 10A,C,E**). In addition, the HAL (L, M, and H dosages) and HAR (H dosage) treatments resulted in an obvious decreasing of Ch and γ-GABA levels compared with the scopolamine-induced group (**Figures 10B,D**). However, different doses of HAL and HAR showed negligible effect on the decrease of L-Phe and L-Tyr induced by scopolamine (**Figures 10F,G**). Generally, the effects of HAL on the important plasmatic and cortical neurotransmitters were more prominent than those of HAR (**Figures 9**, **10**).

## Discussion

Alzheimer’s disease is a deadly progressive neurodegenerative disorder with increasing of aging population and lifetime expectancy, and it is urgent to find new drugs that can effectively treat AD (Kouémou et al., 2017). It is generally known that cholinergic hypofunction, oxidative stress, and neuroinflammation are one of the main characteristics of AD (Abd-El-Fattah et al., 2014; Demirci et al., 2017). Scopolamine is a non-selective antagonist of the muscarinic cholinergic receptor (Jang et al., 2013; Kaur et al., 2017). Numerous studies have shown that scopolamine can impair the ability of learning and memory in humans and animal models, and scopolamine-induced AD model is widely used to study cognitive deficiency and screen for anti-AD drugs (Goverdhan et al., 2012). Scopolamine leads to the cognitive deficits associated with a reduction of cholinergic neurotransmission, increased oxidative stress as well as elevated inflammation in the brain (Lin and Beal, 2006). Accordingly, drugs can efficaciously enhance cholinergic function and/or attenuate oxidative stress and inflammation, and may reverse the learning and memory impairments in scopolamine-induced AD mice, and will ultimately be valuable for the treatment of AD.

Previously, He et al. (2015a) proved that HAR could inhibit the AChE activity and significantly improved spatial learning and memory of cognitively impaired mice, including APP/PS1 transgenic and scopolamine-induced AD mice. In the present study, the memory ameliorating effects of HAL (2, 5, and 10 mg/kg) and HAR (10, 20, and 30 mg/kg) on scopolamine-induced mice were compared using MWM behavioral evaluation. Scopolamine (1 mg/kg) was verified to damage the spatial learning and memory ability of mice in MWM task, which was consistent with our previous studies (He et al., 2015a; Liu et al., 2017). After treatments with HAL (L, M, and H dosages) and HAR (M and H dosages), the escape latency and path length were decreased and the passing frequency were increased effectively (**Figures 2**, **3**), demonstrating that HAL and HAR administrations noticeably improved the learning and spatial memory of the scopolamine-induced mice, and the improvements of HAL were more significant than those of HAR.

In order to further clarify the underlying mechanism for improving learning and memory of HAL and HAR in the scopolamine-induced mice, the alterations of cholinergic system, oxidative stress, and inflammation were assessed. As reported in previous literatures (Abd-El-Fattah et al., 2014; Lin et al., 2016; Demirci et al., 2017), scopolamine i.p. injection led to an attenuation of cholinergic function, elevated oxidative stress and inflammation in current study. Donepezil is a representative symptomatic therapy for mild and moderate AD as an AChE inhibitor (Bohnen et al., 2005), and recent researches have revealed that it also can suppress inflammation and oxidative stress (Saxena et al., 2008; Yoshiyama et al., 2010). In this study, donepezil (positive control) could effectively ameliorate these changes in scopolamine-induced mice and improved their learning and memory. Similar to their activities *in vitro* (Zhao et al., 2013; Liu et al., 2014), HAL and HAR at different dosages not only reversed scopolamine-induced raise of AChE activity, but also resulted in the elevation of neurotransmitter ACh and decrease of Ch in the cerebral cortex, hippocampus or plasma (**Figures 4**, **9**, **10**). Moreover, the protein levels of cortical AChE were evidently inhibited by HAL and HAR (**Figures 6C,D**). As reported by Oh et al. (2009), ChAT-positive neurons were significantly reduced by treatment with scopolamine. The present results indicated that HAL and HAR could reverse the decrease of ChAT activity induced by scopolamine (**Figures 4B,E**), while have no impact on the protein expressions of ChAT (**Figures 6E,F**), and it needs to be further proved. Hence, these confirmed that anti-amnesic effects of HAL and HAR on the cognition dysfunction induced by scopolamine probably associated with modification of cholinergic system (particularly AChE), and HAL demonstrated an obvious cholinergic improvement at low dosage (2 mg/kg).

Additionally, many studies have shown that increased oxidative stress is extensively considered as a prominent contributive risk factor in the development of AD (Ghumatkar et al., 2015; Lu et al., 2018). HAL and HAR might serve as endogenous antioxidants and were found to possess an antioxidant capacity by scavenging free radicals (Tse-Susanna et al., 1991; Berrougui et al., 2006a,b). In this study, the overproduction of ROS (a product of lipid peroxidation, MDA) and reduction of antioxidant enzymes (SOD and GSH-px) were observed in the scopolamine-induced mice (**Figure 5**). However, HAL and HAR administrations observably activated the antioxidant enzymes of SOD and GSH-px and suppressed the formation of MDA in the cortex of the scopolamine-induced dementia mice (**Figure 5**), and then improved the ability of antioxidant defense, indicating the neuroprotective effects of HAL and HAR. Notably, the antioxidative effects of HAL and HAR were generally comparable.

Furthermore, there is growing evidence suggests that memory impairment is associated with increased neuroinflammation (Abd-El-Fattah et al., 2014). According to the study of Bensalem et al. (2014), HAL and HAR demonstrated a significant inhibition on MPO with the IC_50_ of 0.08 and 0.26 μM, respectively. Anti-inflammatory activity of HAR was achieved through inhibiting TNF-α and *NO* in lipopolysaccharide-stimulated mouse RAW264 and human THP-1 cells (Patel et al., 2012). Notably, the high levels of pro-inflammatory cytokines (TNF-α and MPO) and inflammatory mediator (*NO*) were detected in the brain of amnesia mice induced by scopolamine (**Figures 4**, **5**). The current study confirmed that HAL and HAR treatments could simultaneously inhibit the generation of *NO* and the upregulation of TNF-α and MPO, and the inhibitory actions of HAL on TNF-α and *NO* were more apparent than those of HAR (**Figures 4**, **5**). Besides, the results of western blotting and immunofluorescence analysis further evidence that the protein expression levels of MPO were prominently suppressed by HAL and HAR (**Figures 7**, **8**), suggesting that the ability of HAL and HAR to counteract the memory deficits might be mediated by suppressing the inflammatory markers TNF-α, MPO, and *NO*.

As is well-known, AD is associated with inadequate levels of various neurotransmitters (Kumar and Singh, 2015; Svob Strac et al., 2015). Previous studies have reported that mice received scopolamine alone showed a considerably decrease in cholinergic system reactivity, as indicated by a decreased ACh level and an increased AChE activity along with a decreased ChAT activity (Lee et al., 2014). A transgenic animal model of AD demonstrated a falling Glu level (Nilsen et al., 2012), and the serum and plasma metabolomics also indicated a decrease of Trp, 5-HT, Glu, Phe, Tyr in AD model mice or patients (Vermeiren et al., 2014; González-Domínguez et al., 2015; Weng et al., 2015; Corso et al., 2017; Li et al., 2018). Correspondingly, our results proved that the scopolamine-induced dementia was accompanied by the decrease of ACh, L-Trp, 5-HT, L-Glu, L-Phe, L-Tyr and the elevation of Ch and γ-GABA, which is in agreement with the previous studies (Scali et al., 1999; Nilsen et al., 2012; Lee et al., 2014; Vermeiren et al., 2014; González-Domínguez et al., 2015; Kumar and Singh, 2015; Weng et al., 2015; Zhou et al., 2016; Corso et al., 2017; Liu et al., 2017). Neurotransmitters play an important role in the brain circuit involved in many aspects of learning and memory, especially the cholinergic, serotonergic, glutametargic, and GABAergic neurotransmitters (Vermeiren et al., 2014; Kumar and Singh, 2015; Zhou et al., 2016). It is noteworthy that, except for L-Phe and L-Tyr, the other alterations could be effectively reversed by both HAL and HAR administrations in the plasma and cortex, and the modulations of HAL were more striking than those of HAR (**Figures 9**, **10**). Accordingly, the regulations of these neurotransmitters by HAL and HAR may be beneficial to enhance the learning and memory ability of dementia mice.

Generally speaking, HAL (2, 5, and 10 mg/kg) and HAR (20 and 30 mg/kg) showed an enhancement in cholinergic neurotransmission through suppressing AChE and inducing ChAT, enhancing antioxidant defense by increasing SOD and GSH-px activities and reducing MDA production, enhancing anti-inflammatory effects via lowering TNF-α, MPO and *NO*, and modulating the levels of critical neurotransmitters (ACh, Ch, L-Trp, 5-HT, γ-GABA, and L-Glu). Thus, HAL and HAR may act on the multiple targets as the therapeutics for AD. Furthermore, the regulations of HAL on the cholinergic function, inflammation and neurotransmitters were more notable than those of HAR, and HAL showed a similar antioxidant capacity with HAR. Particularly, the effective dosage of HAL (2 mg/kg) was far lower than that of HAR (20 mg/kg), which may be mainly due to the differences in the bioavailability and metabolic stability of the two drugs (Li et al., 2017b). Taken together, all these results revealed that compared to HAR, HAL may be a better candidate compound with superior anti-amnesic and pharmacokinetic characteristics for AD therapy.

## Conclusion

In summary, HAL and HAR could effectively ameliorate memory impairments in a scopolamine-induced mouse model via improvement of cholinergic system function, suppression of oxidative stress and inflammation damage, and modulation of vital neurotransmitters in dementia mice. Especially for HAL, the regulations on the cholinergic function, inflammation and neurotransmitters were more significant than those of HAR, and displayed a comparable antioxidant effect to HAR. Notably, the effective dosage of HAL (2 mg/kg) was far lower than that of HAR (20 mg/kg). The overall results indicate that HAL can be a superior candidate drug for treating learning and memory dysfunctions such as AD. Further evaluations must be undertaken on the anti-amnesic effects and molecular mechanisms of HAL, which will provide substantial initial evidence for the prevention and treatment of AD.

## Author Contributions

S-PL and C-HW: participated in research design, performed data analysis, wrote or contributed to the writing of the manuscript. S-PL, Y-WW, S-LQ, Y-PZ, GD, W-ZD, CM, Q-YL, H-DG, WL, and X-MC: conducted experiments.

## Conflict of Interest Statement

The authors declare that the research was conducted in the absence of any commercial or financial relationships that could be construed as a potential conflict of interest.

## References

[B1] Abd-El-Fattah M. A. Abdelakader N. F. Zaki H. F. (2014). Pyrrolidine dithiocarbamate protects against scopolamine-induced cognitive impairment in rats. *Eur. J. Pharmacol.* 723 330–338. 10.1016/j.ejphar.2013.11.008 24315930

[B2] Aucoin J. S. Jiang P. Aznavour N. Tong X. K. Buttini M. Descarries L. (2005). Selective cholinergic denervation, independent from oxidative stress, in a mouse model of Alzheimer’s disease. *Neuroscience* 132 73–86. 10.1016/j.neuroscience.2004.11.047 15780468

[B3] Bassani T. B. Turnes J. M. Moura E. L. Bonato J. M. Cóppola-Segovia V. Zanata S. M. (2017). Effects of curcumin on short-term spatial and recognition memory, adult neurogenesis and neuroinflammation in a streptozotocin-induced rat model of dementia of Alzheimer’s type. *Behav. Brain Res.* 335 41–54. 10.1016/j.bbr.2017.08.014 28801114

[B4] Bensalem S. Soubhye J. Aldib I. Bournine L. Nguyen A. T. Vanhaeverbeek M. (2014). Inhibition of myeloperoxidase activity by the alkaloids of *Peganum harmala* L. (Zygophyllaceae). *J. Ethnopharmacol.* 154 361–369. 10.1016/j.jep.2014.03.070 24746482

[B5] Berrougui H. Isabelle M. Cloutier M. Hmamouchi M. Khalil A. (2006a). Protective effects of *Peganum harmala* L. extract, harmine and harmaline against human low-density lipoprotein oxidation. *J. Pharm. Pharmacol.* 58 967–974. 10.1211/jpp.58.7.0012 16805957

[B6] Berrougui H. Martín-Cordero C. Khalil A. Hmamouchi M. Ettaib A. Marhuenda E. (2006b). Vasorelaxant effects of harmine and harmaline extracted from *Peganum harmala* L. seed’s in isolated rat aorta. *Pharmacol. Res.* 54 150–157. 10.1016/j.phrs.2006.04.001 16750635

[B7] Bohnen N. I. Kaufer D. I. Hendrickson R. Ivanco L. S. Lopresti B. J. Koeppe R. A. (2005). Degree of inhibition of cortical acetylcholinesterase activity and cognitive effects by donepezil treatment in Alzheimer’s disease. *J. Neurol. Neurosurg. Psychiatry* 76 315–319. 10.1136/jnnp.2004.038729 15716518 PMC1739536

[B8] Bromley-Brits K. Deng Y. Song W. H. (2011). Morris water maze test for learning and memory deficits in Alzheimer’s disease model mice. *J. Vis. Exp.* 53 1–5. 10.3791/2920 21808223 PMC3347885

[B9] Corso G. Cristofano A. Sapere N. La Marca G. Angiolillo A. Vitale M. (2017). Serum amino acid profiles in normal subjects and in patients with or at risk of Alzheimer dementia. *Dement. Geriatr. Cogn. Dis. Extra* 7 143–159. 10.1159/000466688 28626469 PMC5471778

[B10] Coyle J. T. Price D. L. Delong M. R. (1983). Alzheimer’s disease: a disorder of cortical cholinergic innervation. *Science* 219 1184–1190. 10.1126/science.6338589 6338589

[B11] Demirci K. Nazıroğlu M. Övey İ. S. Balaban H. (2017). Selenium attenuates apoptosis, inflammation and oxidative stress in the blood and brain of aged rats with scopolamine-induced dementia. *Metab. Brain Dis.* 32 321–329. 10.1007/s11011-016-9903-1 27631101

[B12] Fan L. Y. Chiu M. J. (2014). Combotherapy and current concepts as well as future strategies for the treatment of Alzheimer’s disease. *Neuropsychiatr. Dis. Treat.* 10 439–451. 10.2147/NDT.S45143 24648738 PMC3956689

[B13] Frison G. Favretto D. Zancanaro F. Fazzin G. Ferrara S. D. (2008). A case of beta-carboline alkaloid intoxication following ingestion of *Peganum harmala* seed extract. *Forensic Sci. Int.* 179 37–43. 10.1016/j.forsciint.2008.05.003 18603389

[B14] Ghumatkar P. J. Patil S. P. Jain P. D. Tambe R. M. Sathaye S. (2015). Nootropic, neuroprotective and neurotrophic effects of phloretin in scopolamine induced amnesia in mice. *Pharmacol. Biochem. Behav.* 135 182–191. 10.1016/j.pbb.2015.06.005 26071678

[B15] González-Domínguez R. García-Barrera T. Gómez-Ariza J. L. (2015). Metabolite profiling for the identification of altered metabolic pathways in Alzheimer’s disease. *J. Pharm. Biomed. Anal.* 107 75–81. 10.1016/j.jpba.2014.10.010 25575172

[B16] Goverdhan P. Sravanthi A. Mamatha T. (2012). Neuroprotective effects of meloxicam and selegiline in scopolamine-induced cognitive impairment and oxidative stress. *Int. J. Alzheimers Dis.* 2012 1–8. 10.1155/2012/974013 22536538 PMC3320018

[B17] Gray E. Thomas T. L. Betmouni S. Betmouni S. Scolding N. Love S. (2008). Elevated activity and microglial expression of myeloperoxidase in demyelinated cerebral cortex in multiple sclerosis. *Brain Pathol.* 18 86–95. 10.1111/j.1750-3639.2007.00110.x 18042261 PMC8095620

[B18] He D. D. Wu H. Wei Y. Liu W. Huang F. Shi H. L. (2015a). Effects of harmine, an acetylcholinesterase inhibitor, on spatial learning and memory of APP/PS1 transgenic mice and scopolamine-induced memory impairment mice. *Eur. J. Pharmacol.* 768 96–107. 10.1016/j.ejphar.2015.10.037 26526348

[B19] He D. D. Zhang L. Liu L. Wu X. J. Cheng X. M. Wang C. H. (2015b). Total alkaloids from the seeds of *Peganum harmala* ameliorating mice learning ability and memory. *Chin. Tradit. Pat. Med.* 37 478–482.

[B20] Heneka M. T. Carson M. J. El Khoury J. Landreth G. E. Brosseron F. Feinstein D. L. (2015). Neuroinflammation in Alzheimer’s disease. *Lancet Neurol.* 14 388–405. 10.1016/S1474-4422(15)70016-5 25792098 PMC5909703

[B21] Herraiz T. Gonzalez D. Ancin-Azpilicueta C. Arán V. J. Guillén H. (2010). Beta-carboline alkaloids in *Peganum harmala* and inhibition of human monoamine oxidase (MAO). *Food Chem. Toxicol.* 48 839–845. 10.1016/j.fct.2009.12.019 20036304

[B22] Jang Y. J. Kim J. Shim J. Kim C. Y. Jang J. H. Lee K. W. (2013). Decaffeinated coffee prevents scopolamine-induced memory impairment in rats. *Behav. Brain Res.* 245 113–119. 10.1016/j.bbr.2013.02.003 23415910

[B23] Jeon S. J. Kim B. Park H. J. Zhang J. B. Kwon Y. Kim D. H. (2017). The ameliorating effect of 1-palmitoyl-2-linoleoyl-3-acetylglycerol on scopolamine-induced memory impairment via acetylcholinesterase inhibition and LTP activation. *Behav. Brain Res.* 324 58–65. 10.1016/j.bbr.2017.01.040 28137622

[B24] Jiang B. (2016). *Study on the Transportation of Alkaloids from Peganum harmala through the Blood Brain Barrier and the Mechanism of Their Effect on the Neurotransmitters in Rats Brain.* Doctor dissertation, Shanghai University of Traditional Chinese Medicine, Shanghai.

[B25] Jiménez J. Riveron-Negrete L. Abdullaev F. Espinosa-Aguirrec J. Rodríguez-Arnaiza R. (2008). Cytotoxicity of the β-carboline alkaloids harmine and harmaline in human cell assays in vitro. *Exp. Toxicol. Pathol.* 60 381–389. 10.1016/j.etp.2007.12.003 18430551

[B26] Kaur R. Singh V. Shri R. (2017). Anti-amnesic effects of Ganoderma species: a possible cholinergic and antioxidant mechanism. *Biomed. Pharmacother.* 92 1055–1061. 10.1016/j.biopha.2017.06.029 28618650

[B27] Kouémou N. E. Taiwe G. S. Moto F. C. O. Pale S. Gwladys N. T. Njapdounke J. S. K. (2017). Nootropic and neuroprotective effects of dichrocephala integrifolia on scopolamine mouse model of Alzheimer’s disease. *Front. Pharmacol.* 8:847 10.3389/fphar.2017.00847 PMC5702348 29209218

[B28] Kumar A. Singh A. (2015). A review on Alzheimer’s disease pathophysiology and its management: an update. *Pharmacol. Rep.* 67 195–203. 10.1016/j.pharep.2014.09.004 25712639

[B29] Kwon S. H. Lee H. K. Kim J. A. Hong S. I. Kim H. C. Jo T. H. (2010). Neuroprotective effects of chlorogenic acid on scopolamine-induced amnesia via anti-acetylcholinesterase and anti-oxidative activities in mice. *Eur. J. Pharmacol.* 649 210–217. 10.1016/j.ejphar.2010.09.001 20854806

[B30] Lee H. E. Jeon S. J. Ryu B. Park S. J. Ko S. Y. Lee Y. (2016). Swertisin, a C-glucosylflavone, ameliorates scopolamine-induced memory impairment in mice with its adenosine A1 receptor antagonistic property. *Behav. Brain Res.* 306 137–145. 10.1016/j.bbr.2016.03.030 26996316

[B31] Lee S. Kim J. Seo S. G. Choi B. R. Han J. S. Lee K. W. (2014). Sulforaphane alleviates scopolamine-induced memory impairment in mice. *Pharmacol. Res.* 85 23–32. 10.1016/j.phrs.2014.05.003 24836869

[B32] Li J. Liu Y. Li W. Wang Z. Guo P. Li L. (2018). Metabolic profiling of the effects of ginsenoside Re in an Alzheimer’s disease mouse model. *Behav. Brain Res.* 337 160–172. 10.1016/j.bbr.2017.09.027 28927718

[B33] Li S. P. Cheng X. M. Wang C. H. (2017a). A review on traditional uses, phytochemistry, pharmacology, pharmacokinetics and toxicology of the genus *Peganum*. *J. Ethnopharmacol.* 203 127–162. 10.1016/j.jep.2017.03.049 28359849

[B34] Li S. P. Zhang Y. P. Deng G. Wang Y. W. Qi S. L. Cheng X. M. (2017b). Exposure characteristics of the analogous β-carboline alkaloids harmaline and harmine based on the efflux transporter of multidrug resistance protein 2. *Front. Pharmacol.* 8:541 10.3389/fphar.2017.00541 PMC5566973 28871225

[B35] Lin J. J. Huang L. Yu J. Xiang S. Y. Wang J. L. Zhang J. R. (2016). Fucoxanthin, a marine carotenoid, reverses scopolamine-induced cognitive impairments in mice and inhibits acetylcholinesterase in vitro. *Mar. Drugs* 14:67. 10.3390/md14040067 27023569 PMC4849071

[B36] Lin M. T. Beal M. F. (2006). Mitochondrial dysfunction and oxidative stress in neurodegenerative diseases. *Nature* 443 787–795. 10.1038/nature05292 17051205

[B37] Liu W. Yang Y. D. Cheng X. M. Gong C. Li S. P. He D. D. (2014). Rapid and sensitive detection of the inhibitive activities of acetyl- and butyryl-cholinesterases inhibitors by UPLC-ESIMS/MS. *J. Pharm. Biomed. Anal.* 94 215–220. 10.1016/j.jpba.2014.02.004 24631841

[B38] Liu W. Zhu Y. D. Wang Y. L. Qi S. L. Wang Y. W. Ma C. (2017). Anti-amnesic effect of extract and alkaloid fraction from aerial parts of *Peganum harmala* on scopolamine-induced memory deficits in mice. *J. Ethnopharmacol.* 204 95–106. 10.1016/j.jep.2017.04.019 28442406

[B39] Louis E. D. Jiang W. Gerbin M. Mullaney M. M. Zheng W. (2010). Relationship between blood harmane and harmine concentrations in familial essential tremor, sporadic essential tremor and controls. *Neurotoxicology* 31 674–679. 10.1016/j.neuro.2010.08.003 20708029 PMC2974038

[B40] Lu C. Dong L. M. Lv J. W. Wang Y. Fan B. Wang F. (2018). 20(S)-protopanaxadiol (PPD) alleviates scopolamine-induced memory impairment via regulation of cholinergic and antioxidant systems, and expression of Egr-1, c-Fos and c-Jun in mice. *Chem. Biol. Interact.* 279 64–72. 10.1016/j.cbi.2017.11.008 29133030

[B41] Mahmoudian M. Jalipour H. Dardashti P. S. (2002). Toxicity of *Peganum harmala*: review and a case report. *Iran. J. Pharmacol. Ther.* 1 1–4.

[B42] Malik J. Kaur J. Choudhary S. (2017). Standardized extract of Lactuca sativa Linn. and its fractions abrogates scopolamine-induced amnesia in mice: a possible cholinergic and antioxidant mechanism. *Nutr. Neurosci.* 1–12. 10.1080/1028415X.2017.1291166 [Epub ahead of print]. 28245707

[B43] Morris R. G. Garrud P. Rawlins J. A. O’Keefe J. (1982). Place navigation impaired in rats with hippocampal lesions. *Nature* 297 681–683. 10.1038/297681a0 7088155

[B44] Nilsen L. H. Melø T. M. Sæther O. Witter M. P. Sonnewald U. (2012). Altered neurochemical profile in the McGill-R-Thy1-APP rat model of Alzheimer’s disease: a longitudinal in vivo 1H MRS study. *J. Neurochem.* 123 532–541. 10.1111/jnc.12003 22943908

[B45] Oh J. H. Choi B. J. Chang M. S. Park S. K. (2009). Nelumbo nucifera semen extract improves memory in rats with scopolamine-induced amnesia through the induction of choline acetyltransferase expression. *Neurosci. Lett.* 461 41–44. 10.1016/j.neulet.2009.05.045 19463889

[B46] Patel K. Gadewar M. Tripathi R. Prasad S. K. Patel D. K. (2012). A review on medicinal importance, pharmacological activity and bioanalytical aspects of beta-carboline alkaloid “Harmine”. *Asian Pac. J. Trop. Biomed.* 2 660–664. 10.1016/S2221-1691(12)60116-6 23569990 PMC3609365

[B47] Rijpma A. Meulenbroek O. Rikkert M. O. (2014). Cholinesterase inhibitors and add-on nutritional supplements in Alzheimer’s disease: a systematic review of randomized controlled trials. *Ageing Res. Rev.* 16 105–112. 10.1016/j.arr.2014.06.002 24982004

[B48] Saxena G. Singh S. P. Agrawal R. Nath C. (2008). Effect of donepezil and tacrine on oxidative stress in intracerebral streptozotocin-induced model of dementia in mice. *Eur. J. Pharmacol.* 581 283–289. 10.1016/j.ejphar.2007.12.009 18234183

[B49] Scali C. Prosperi C. Giovannelli L. Bianchi L. Pepeu G. Casamenti F. (1999). β (1-40) Amyloid peptide injection into the nucleus basalis of rats induces microglia reaction and enhances cortical γ-aminobutyric acid release in vivo. *Brain Res.* 831 319–321. 10.1016/S0006-8993(99)01492-4 10412015

[B50] Shi X. Y. Liu W. Zhang L. Li S. P. Cheng X. M. Xi Y. (2014). Pharmacokinetics of harmaline, harmine and their metabolites in rats administered with total alkaloid extracts from *Peganum harmala* L. *Chin. Tradit. Pat. Med.* 36 1169–1175. 10.3969/j.issn.1001-1528.2014.06.013

[B51] Singh S. Kaur H. Sandhir R. (2016). Fractal dimensions: a new paradigm to assess spatial memory and learning using Morris water maze. *Behav. Brain Res.* 299 141–146. 10.1016/j.bbr.2015.11.023 26592165

[B52] Svob Strac D. Muck-Seler D. Pivac N. (2015). Neurotransmitter measures in the cerebrospinal fluid of patients with Alzheimer’s disease: a review. *Psychiatr. Danub.* 27 14–24. 25751428

[B53] Tse-Susanna Y. H. Mak I. T. Dickens B. F. (1991). Antioxidative properties of harmane and β-carboline alkaloids. *Biochem. Pharmacol.* 42 459–464. 10.1016/0006-2952(91)90305-O 1859459

[B54] Vermeiren Y. Van Dam D. Aerts T. Engelborghs S. De Deyn P. P. (2014). Monoaminergic neurotransmitter alterations in postmortem brain regions of depressed and aggressive patients with Alzheimer’s disease. *Neurobiol. Aging* 35 2691–2700. 10.1016/j.neurobiolaging.2014.05.031 24997673

[B55] Verri M. Pastoris O. Dossena M. Aquilani R. Guerriero F. Cuzzoni G. (2012). Mitochondrial alterations, oxidative stress and neuroinflammation in Alzheimer’s disease. *Int. J. Immunopathol. Pharmacol.* 25 345–353. 10.1177/039463201202500204 22697066

[B56] Wang C. H. Zhang Z. X. Wang Y. H. He X. J. (2015). Cytotoxic indole alkaloids against human leukemia cell lines from the toxic plant *Peganum harmala*. *Toxins* 7 4507–4518. 10.3390/toxins7114507 26540074 PMC4663518

[B57] Weng R. Shen S. S. Tian Y. L. Burton C. Xu X. Y. Liu Y. (2015). Metabolomics approach reveals integrated metabolic network associated with serotonin deficiency. *Sci. Rep.* 5:11864. 10.1038/srep11864 26154191 PMC4495385

[B58] Yoshiyama Y. Kojima A. Ishikawa C. Arai K. (2010). Anti-inflammatory action of donepezil ameliorates tau pathology, synaptic loss, and neurodegeneration in a tauopathy mouse mode. *J. Alzheimers Dis.* 22 295–306. 10.3233/JAD-2010-100681 20847440

[B59] Zhang L. (2013). *Study on the Film Coating Tablets of Total Alkaloids from Peganum harmala.* Master Dissertation, Shanghai University of Traditional Chinese Medicine, Shanghai.

[B60] Zhao T. Ding K. M. Zhang L. Cheng X. M. Wang C. H. Wang Z. T. (2013). Acetylcholinesterase and butyrylcholinesterase inhibitory activities of β-carboline and quinoline alkaloids derivatives from the plants of genus *Peganum*. *J. Chem.* 2013:717232 10.1155/2013/717232

[B61] Zhou X. L. Li Y. F. Shi X. Z. Ma C. (2016). An overview on therapeutics attenuating amyloid β level in Alzheimer’s disease: targeting neurotransmission, inflammation, oxidative stress and enhanced cholesterol levels. *Am. J. Transl. Res.* 8 246–269. 27158324 PMC4846881

